# Akt Activation is Required for TGF-β1-Induced Osteoblast Differentiation of MC3T3-E1 Pre-Osteoblasts

**DOI:** 10.1371/journal.pone.0112566

**Published:** 2014-12-03

**Authors:** Eiichi Suzuki, Hiromi Ochiai-Shino, Hideto Aoki, Shoko Onodera, Akiko Saito, Atsushi Saito, Toshifumi Azuma

**Affiliations:** 1 Department of Periodontology, Tokyo Dental College, Tokyo, Japan; 2 Department of Biochemistry, Tokyo Dental College, Tokyo, Japan; 3 Oral Health Science Center, Tokyo Dental College, Tokyo, Japan; University of Texas Southwestern Medical Center, United States of America

## Abstract

**Background:**

We have previously reported that repeated treatment of human periodontal ligament cells and murine pre-osteoblast MC3T3-E1 cells with transforming growth factor-beta 1 (TGF-β1) inhibited their osteoblastic differentiation because of decreased insulin-like growth factor-1 (IGF-1) secretion. We also found that IGF-1/PI3K signaling plays an important role in osteoblast differentiation induced by TGF-β1 treatment; however, the downstream signaling controlling this remains unknown. The aim of this current study is to investigate whether Akt activation is required for osteoblast differentiation.

**Methodology/Principal Findings:**

MC3T3-E1 cells were cultured in osteoblast differentiation medium (OBM) with or without 0.1 ng/mL TGF-β1. OBM containing TGF-β1 was changed every 12 h to provide repeated TGF-β1 administration. MC3T3-E1 cells were infected with retroviral vectors expressing constitutively active (CA) or dominant-negative (DN)-Akt. Alkaline phosphatase (ALP) activity and osteoblastic marker mRNA levels were substantially decreased by repeated TGF-β1 treatment compared with a single TGF-β1 treatment. However, expression of CA-Akt restored ALP activity following TGF-β1 treatment. Surprisingly, ALP activity increased following multiple TGF-β1 treatments as the number of administrations of TGF-β1 increased. Activation of Akt significantly enhanced expression of osteocalcin, but TGF-β1 treatment inhibited this. Mineralization of MC3T3-E1 cells was markedly enhanced by CA-Akt expression under all medium conditions. Exogenous IGF-1 restored the down-regulation of osteoblast-related gene expression by repeated TGF-β1 administration. However, in cells expressing DN-Akt, these levels remained inhibited regardless of IGF-1 treatment. These findings indicate that Akt activation is required for the early phase of osteoblast differentiation of MC3T3-E1 cells induced by TGF-β1. However, Akt activation is insufficient to reverse the inhibitory effects of TGF-β1 in the late stages of osteoblast differentiation.

**Conclusions:**

TGF-β1 could be an inducer or an inhibitor of osteoblastic differentiation of MC3T3-E1 cells depending on the state of Akt phosphorylation. Our results indicate that Akt is the molecular switch for TGF-β1-induced osteoblastic differentiation of MC3T3-E1 cells.

## Introduction

Inflammatory periodontal disease is the major cause of tooth loss in adults [1]. Regeneration of tooth-supporting tissues including alveolar bone is the ultimate goal for treatment of periodontal diseases [2]. Many preclinical and clinical studies have indicated that the use of growth factors could be a viable treatment modality for periodontal regeneration. Indeed, local application of platelet-derived growth factor (PDGF)-BB, fibroblast growth factor-2 (FGF-2), or bone morphogenetic proteins (BMPs) has demonstrated encouraging results [3]. Other potential approaches to enhancing periodontal regeneration such as stem cell treatment and gene therapy also have drawn much attention [4].

Transforming growth factor (TGF)-βl influences a wide variety of important cellular activities and is secreted by a diverse range of cells that include immune cells localizing to inflammatory sites [5]. Importantly, TGF-β1 can stimulate osteoblast proliferation and regulate osteoclast functions, such as the production and secretion of osteoclast Wnt10b, and could contribute to coupling [6]. Therefore, TGF-β1 has potential as a promising candidate for the treatment of periodontal diseases. However, recent studies have revealed that TGF-β1 is a pivotal modulator of connective tissue regeneration and bone remodeling [7]. Here, TGF-β1 induces differentiation and proliferation of osteoblasts and their precursors, with the precise response dependent on the cell phenotype and stage of maturity [8]–[10]. TGF-β1 also increases alkaline phosphatase (ALP) activity in murine bone marrow stromal cells [11]. Although TGF-β1 promotes osteoblast differentiation and bone formation [12]–[15], it inhibits osteogenesis by various mechanisms depending on its concentration, the cell density, and the differentiation stage of the target cells [16]–[18].

A major pathway by which TGF-β1 exerts its various effects on cells is via phosphatidylinositol 3-kinases (PI3K) signaling. PI3K is a central signaling molecule that plays important roles in many cellular activities [19]–[21]. PI3K phosphorylates PIP_2_ to PIP_3_ within the membrane, enabling the interaction of PIP_3_ with the GTP-binding proteins Rac, PKC, or Akt. Akt in particular has been studied as the major target of PI3K signaling, and the PI3K/Akt pathway can be activated by growth factors and other extracellular signals to regulate many fundamental cellular processes including cell growth, proliferation, and survival [19], [22], [23]. Several signal transduction pathways including Smad signaling and the mitogen-activated protein kinase (MAPK) cascade have been implicated in the formation of bone [24], and recent reports indicate that the PI3K-Akt signaling pathway could be important for osteoblast differentiation [25]–[29]. However, the role of the PI3K pathway in TGF-β1-induced osteoblast differentiation remains unknown.

Previously, we have reported that repeated TGF-β1 treatment inhibited osteoblastic differentiation of human periodontal ligament (HPDL) cells via suppression of insulin-like growth factor-1 (IGF-1) expression [30]. The PI3K/Akt pathway is activated by the IGF-I receptor via the adaptor molecules insulin receptor substrate (IRS)-l and IRS-2 in bone cells [31], [32], and PI3K signaling has been found to have an essential role in IGF-induced osteoblast differentiation [20]. Therefore, persistent exposure to TGF-β1 could inhibit osteoblast differentiation via the suppression of IGF-1 expression and subsequent down-regulation of the PI3K/Akt pathway.

Here, we have investigated the functional roles of Akt in TGF-β1-regulated osteogenesis. We found that repeated TGF-β1 treatment reduced ALP activity in MC3T3-E1 cells. However, forced expression of constitutively active (CA)-Akt ameliorated this suppression of ALP activity under conditions of repeated TGF-β1 administration. While ALP activity was increased following TGF-β1 treatment, *osteocalcin* mRNA levels were decreased. We conclude that Akt activation is a critical step in the TGF-β1-induced osteoblast differentiation of murine pre-osteoblast cells, but its functional role differs depending on the phase of osteogenesis.

## Materials and Methods

### Cell culture

MC3T3-E1 cells were purchased from RIKEN BioResource Center (Ibaraki, Japan) and cultured in α-MEM (Invitrogen, Carlsbad, CA, USA) containing 10% fetal bovine serum (FBS) and 1% penicillin and streptomycin. The Platinum-E Retroviral Packaging Cell Line (PLAT-E; Cell Biolabs, San Diego, CA, USA) was maintained in Dulbecco's modified Eagle medium (DMEM; Sigma-Aldrich, St. Louis, MO, USA), 10% FBS, 1 µg/mL puromycin, 10 µg/mL blasticidin, 1% penicillin and streptomycin.

### Retroviral Gene Transfer and Osteogenic Differentiation

PLAT-E cells (3.5×10^6^ cells/100 mm dish) were plated in DMEM and incubated overnight. Cells were transfected with 9 µg of relevant plasmid DNA (pBabe-puro; Mock, pBabe-puro-AKT1; CA-Akt, pBabe-puroL-myr-Akt; DN-Akt) using Fugene 6 transfection reagent (Roche Diagnostics, Basel, Switzerland). The following day, MC3T3-E1 cells were seeded at 1.2×10^5^ cells/cm^2^ and incubated overnight. Forty-eight hours after transfection, the culture medium was replaced with equal amounts of PLAT-E supernatant containing each of the three retroviruses, which were collected and filtered through a 0.45-µm filter supplemented with 4 µg/mL of polybrene, and incubated overnight. The next day cells were detached and reseeded at 2×10^5^ cells/cm^2^. Subsequently, 24 h later the medium was changed to osteoblast differentiation medium (OBM), consisting of α-MEM supplemented with 50 µg/mL L-ascorbic acid (Wako Pure Chemical Industries Ltd., Osaka, Japan) and 10 mM β-glycerophosphate, with or without 0.1 ng/mL recombinant human (rh) TGF-β1 (Wako Pure Chemical Industries Ltd., Osaka, Japan) as indicated. This concentration of TGF-β1 was optimized in our previous study [30] and preliminary experiments. Under the condition of a single instance of TGF-β1 administration, the medium was not changed until day three. Otherwise, culture media were replaced with new media containing fresh TGF-β1 every 12 h. The cells were also treated with or without 5, 10, or 25 µM PI3K inhibitor LY294002 (Cell Signaling Technology Inc., Danvers, MA, USA) and 200 ng/mL rhIGF-1 (Wako Pure Chemical Industries Ltd., Osaka, Japan) as indicated.

### ALP Activity and Mineralization

Three days after treatment, cells were washed twice with phosphate-buffered saline (PBS), fixed in 4% paraformaldehyde for 5 min at room temperature, and then washed three more times with PBS. For staining, an ALP substrate solution (Roche Diagnostics, Basel, Switzerland) was added to fixed cells for 30 min at room temperature. Cells were then washed three times with distilled water, and images were scored. ALP activity was quantitatively measured as follows. The cells were washed twice with PBS and lysed with lysis buffer (10 mM Tris-HCl (pH 7.5), 150 mM NaCl, complete protease inhibitor mixture, and 1% Nonidet P-40). The protein concentration was measured with a DC protein assay kit (Bio-Rad, Marnes-la-Coquette, France) according to the manufacturer's instructions. ALP activity was assayed using *p*-nitrophenyl phosphate as a substrate, and calculated as micromoles of *p*-nitrophenol/min/mg of protein.

To detect calcium deposits in mineralized tissue, cells were fixed by the same method described above and then stained with Alizarin Red S solution (pH 6.38; Wako Pure Chemical Industries Ltd., Osaka, Japan) for 5 min at room temperature.

### Quantitative Real Time-PCR (qRT-PCR)

We measured the expression of osteoblast differentiation markers by qRT-PCR. Total RNA was extracted using QIAzol reagent (Qiagen Inc., Valencia, CA, USA) according to the manufacturer's instructions. cDNA was synthesized using a high capacity cDNA reverse transcription kit (Applied Biosystems, Foster City, CA, USA). qRT-PCR analysis was performed using the Premix Ex Taq reagent (Takara Bio Inc., Shiga, Japan) according to the manufacturer's instructions. Target genes included *Alp*, *Igf-1*, bone sialoprotein (*Bsp*), osteocalcin (*Oc*), runt-related transcription factor 2 (*Runx2*), and osterix (*Osx*). Measurement of 18S rRNA served as an internal control. All primers and probes are presented in Table 1 and were designed using Probefinder Version 2.45. The relative levels of gene expression were estimated using the 2^−ΔΔ*Ct*^ method.

**Table 1:** Primers used for qRT-PCR.

	Gen Bank	Forward primer	Reverse primer
Gene symbol	accession no.	sequence	sequence
*Alp*	NM_007431.2	cggatcctgaccaaaaacc	tcatgatgtccgtggtcaat
*Bsp*	NM_008318.2	gaaaatggagacggcgatag	cattgttttcctcttcgtttga
*Igf-1*	NM_001111274.1	tcggcctcatagtacccact	acgacatgatgtgtatctttattgc
*Oc*	NM_007541.2	agactccggcgctacctt	ctcgtcacaagcagggttaag
*Osx (Sp7)*	NM_130458.3	ctcctgcaggcagtcctc	gggaagggtgggtagtcatt
*Runx2*	NM_001145920.1	gcccaggcgtatttcaga	tgcctggctcttcttactgag
18S *rRNA*	M11188.1	cggacaggattgacagattg	cgctccaccaactaagaacg

### Protein Extraction and Immunoblotting

Cells were lysed with lysis buffer (10 mM Tris-HCl (pH 7.5), 150 mM NaCl, complete protease inhibitor mixture, 1 mM sodium orthovanadate, and 1% Nonidet P-40), and the protein content was measured using a DC protein assay kit (Bio-Rad, Marnes-la-Coquette, France). Equivalent amounts of protein were separated by electrophoresis on NuPAGE 4–12% Bis-Tris gels (Invitrogen) and transferred to a PVDF membrane. The membranes were probed with anti-Akt (1∶1000; #4691, Cell Signaling Technology Inc.) and anti-phosphorylated Akt (1∶2000; #4060, Cell Signaling Technology Inc.) antibodies, followed by HRP-conjugated goat anti-rabbit IgG. Bound antibodies were visualized using a chemiluminescent substrate (ECL plus; GE Healthcare, Buckinghamshire, UK) and ImageQuant LAS 4000 mini (GE Healthcare).

### Statistical Analysis

All data are expressed as mean ± standard error (S.E.). When analysis of variance indicated differences between groups, multiple comparisons between each experimental group were performed using the Bonferroni test. Statistical significance was defined as *p*<0.05.

## Results

### PI3K inhibition and repeated TGF-β1 treatment inhibited osteoblast differentiation in MC3T3-E1 cells

**Figure 1:**
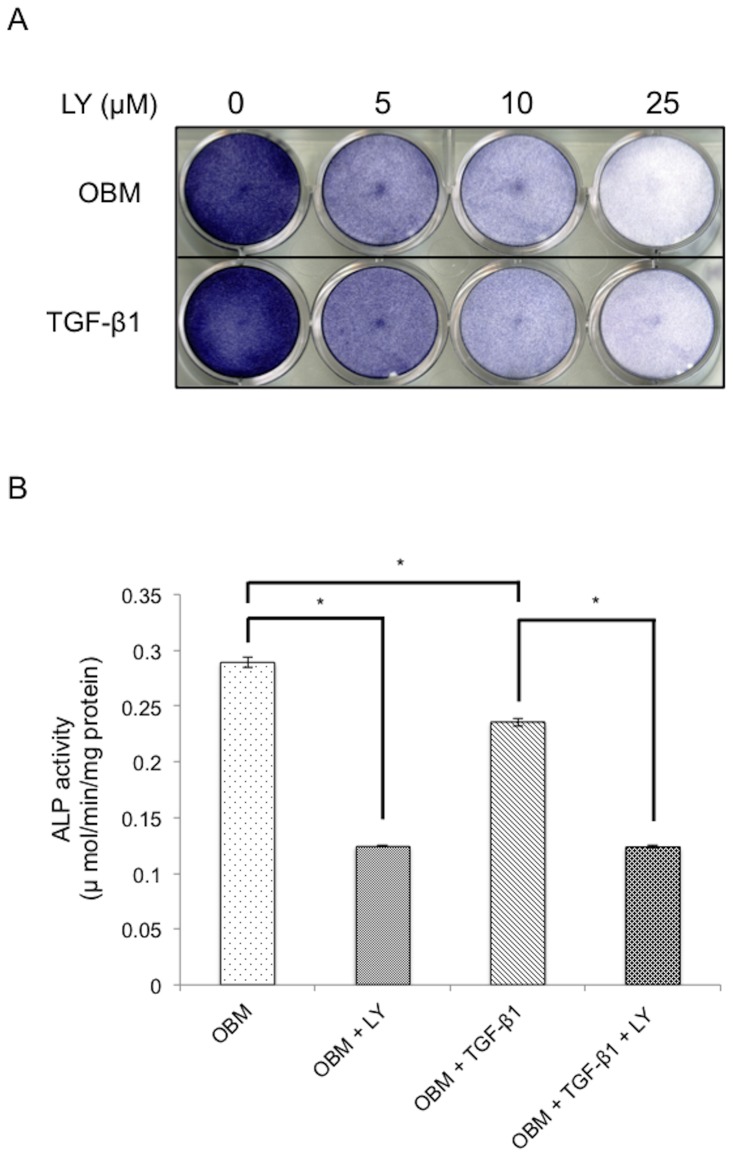
Abrogation of PI3K signaling inhibits TGF-β1-mediated osteoblast differentiation in MC3T3-E1 cells. (A) Confluent MC3T3-E1 cells were cultured in OBM with and without 0.1 ng/mL TGF-β1 in the absence or presence of the PI3K inhibitor LY294002 (5, 10, or 25 µM) for 72 h. ALP activity was visualized by ALP staining of cells. (B) Cells were cultured in OBM with and without 0.1 ng/mL TGF-β1 in the absence or presence of 10 µM LY294002 for 72 h, and ALP activity was measured. Each experiment was performed in triplicate, and the data represent the means ± S.E. (*n* = 3). The Bonferroni correction for multiple comparisons was applied. *, *p*<0.001.

**Figure 2:**
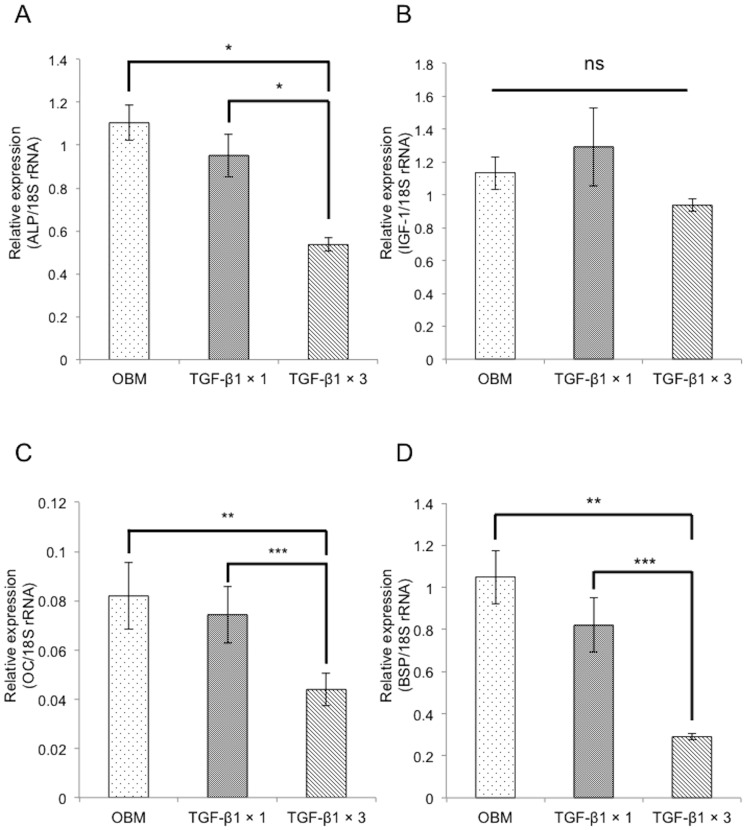
Repeated TGF-β1 treatment inhibits expression of osteoblast differentiation markers in MC3T3-E1 cells. Confluent MC3T3-E1 cells were treated for 72 h with OBM, OBM with a single administration of 0.1 ng/mL TGF-β1, and OBM with a triple administration of 0.1 ng/mL TGF-β1. Repeated TGF-β1 treatment significantly decreased the expression of *Alp* (A), *Oc* (C), and *Bsp* (D). The decrease in the expression of *Igf-1* (B) was not statistically significant. Expression of these genes was analyzed by qRT-PCR, and their mRNA levels were normalized to that of 18S rRNA and measured in triplicate. Values represent mean ± S.E. (*n* = 3). Bonferroni correction for multiple comparisons was applied. *, *p*<0.001; **, *p*<0.01; ***, *p*<0.05.

We have previously shown that repeated administration of TGF-β1 inhibits osteoblastic differentiation of HPDL cells through suppression of IGF-1 expression and the subsequent down-regulation of the PI3K/Akt pathway [30]. It has been well established that TGF-β1 inhibits osteoblastic differentiation of MC3T3-E1 cells. Here, we have built upon these findings by investigating whether inhibition of PI3K could suppress ALP activity. The ALP activity of cells in OBM containing the PI3K inhibitor LY294002 was decreased regardless of the presence of TGF-β1 (Fig. 1A and B). We also found that repeated administration of 0.1 ng/mL TGF-β1 resulted in decreased mRNA expression of osteoblast differentiation-related genes such as *Alp*, *Oc*, and *Bsp* (Fig. 2A, C, D), although the decrease in *Igf-1* (Fig. 2B) was not statistically significant.

### Overexpression of constitutively active Akt reversed the inhibition of ALP activity mediated by repeated TGF-β1 administration

**Figure 3:**
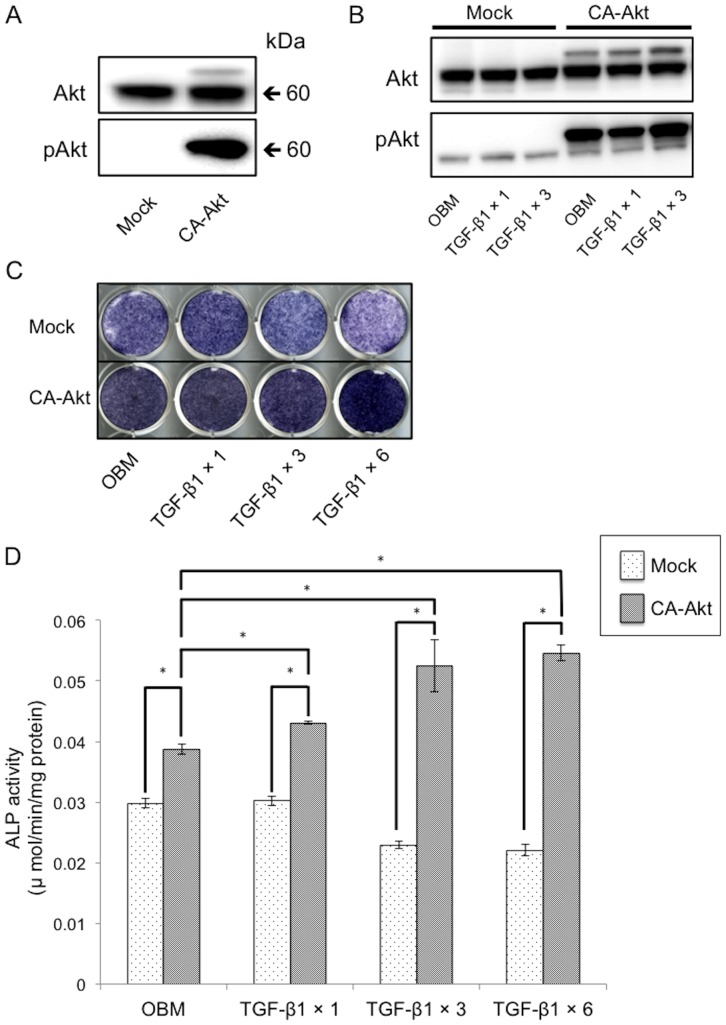
Overexpression of CA-Akt reverses the inhibition of ALP activity induced by repeated administration of TGF-β1. MC3T3-E1 cells were infected with CA-Akt vector or Mock vector. Phosphorylation of Akt is evident in CA-Akt cells cultured in α-MEM (A). Then cells were treated with or without repeated administration of 0.1 ng/mL TGF-β1 for 72 h in OBM. The levels of phosphorylated Akt in these cells were detected by western blot analysis (B). ALP staining (C) and ALP activity (D) were assessed following repeated TGF-β1 administration. Figures shown represent at least three independent experiments. Values represent mean ± S.E. (*n* = 4). Bonferroni correction for multiple comparisons was applied. *, *p*<0.001.

MC3T3-E1 cells were infected with mock retrovirus or that encoding CA-Akt (Fig. 3A). In MC3T3-E1 cells infected with the mock virus (Mock cells), a single dose of TGF-β1 increased pAkt, while repeated TGF-β1 administration decreased pAkt following the third treatment (Fig. 3B). However, in MC3T3-E1 cells expressing CA-Akt (CA-Akt cells), pAkt was significantly increased after this third treatment. In Mock cells, ALP activity was significantly decreased as the number of administrations of TGF-β1 increased, while the opposite trend was found for CA-Akt cells (Fig. 3C and D).

### Forced expression of dominant-negative Akt abrogated the effect of IGF-1

**Figure 4:**
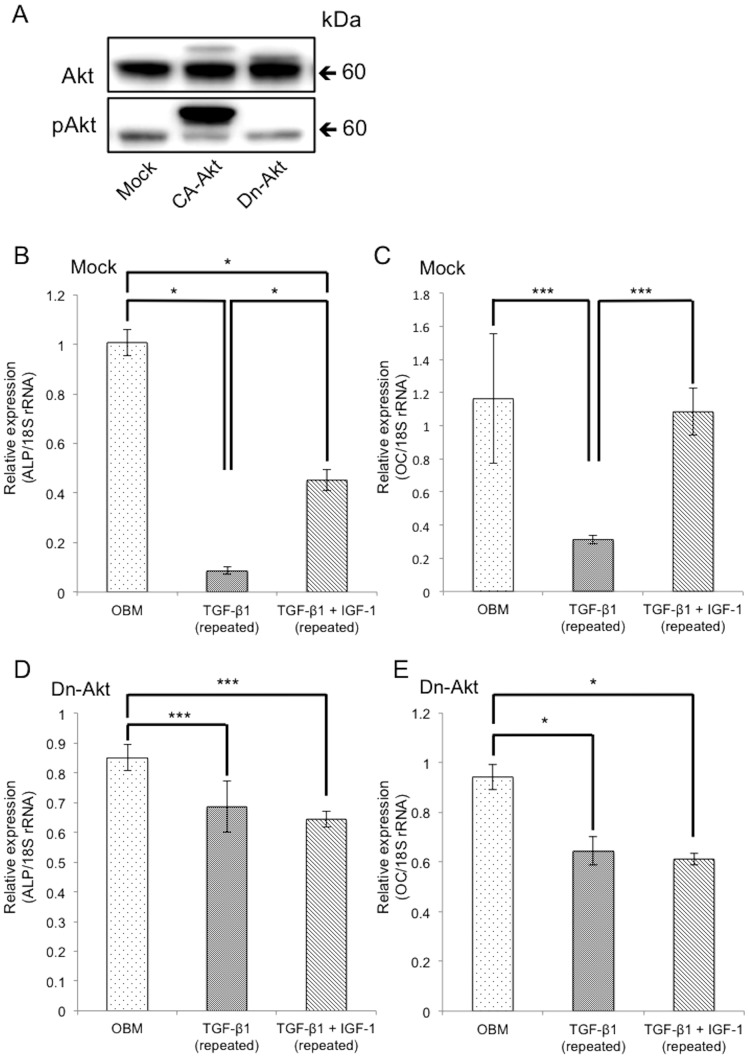
Forced expression of dominant-negative Akt abrogates the pro-differentiation effect of IGF-1. MC3T3-E1 cells were infected with DN-Akt vector or Mock vector (A), and then cells were cultured in OBM with and without a triple administration of 0.1 ng/mL TGF-β1 for 72 h. Exogenous IGF-1 (200 ng/mL) was applied in conjunction with administration of TGF-β1 and the expression of *Alp* and *Oc* mRNA levels measured in Mock (B, C) and DN-Akt (D, E) cells. Gene expression was analyzed by qRT-PCR, and mRNA levels were normalized to that of 18S rRNA and measured in triplicate. Values represent mean ± S.E. (*n* = 3). Bonferroni correction for multiple comparisons was applied. *, *p*<0.001; ***, *p*<0.05.

We have previously demonstrated that exogenous IGF-1 recovered the suppression of osteoblast differentiation induced by repeated TGF-β1 treatment [30]. Here, we have further investigated the effect of IGF-1 on osteoblastic differentiation of cells expressing dominant-negative (DN)-Akt (Fig. 4A). In Mock cells, the expression of *Alp* and *Oc* was significantly decreased by repeated TGF-β1 treatment, while the addition of 200 ng/mL IGF-1 partially reversed this inhibition (Fig. 4B and C). In MC3T3-E1 cells expressing DN-Akt (DN-Akt cells), however, the addition of IGF-1 exerted no significant effect on the expression of *Alp* and *Oc* induced by TGF-β1 treatment (Fig. 4D and E).

### Overexpression of CA-Akt enhanced expression of osteoblast differentiation markers (Osx, Oc) and mineralization, but could not prevent TGF-β1-mediated inhibition of osteoblast differentiation

**Figure 5:**
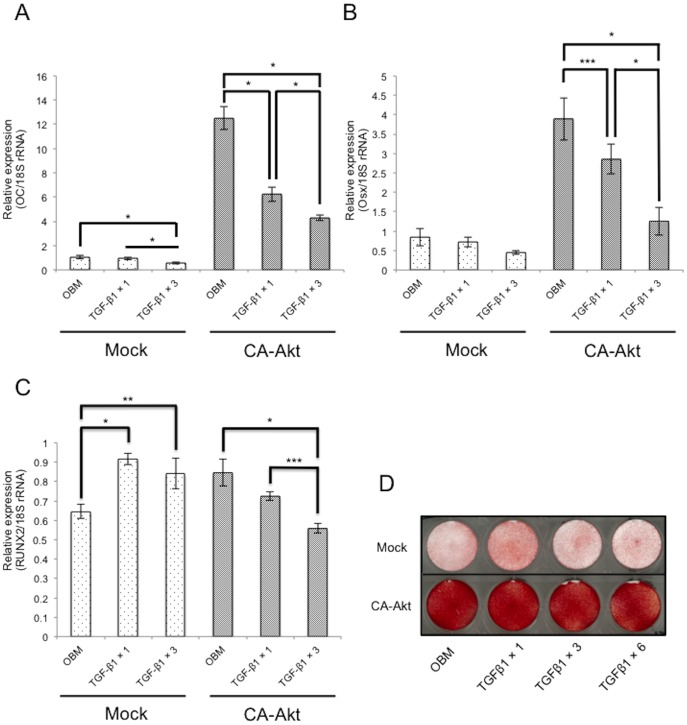
CA-Akt does not prevent inhibition of *Osx* and *Oc* expression by TGF-β1. MC3T3-E1 cells were infected with CA-Akt vector or Mock vector, and then cells were treated with or without repeated administration of 0.1 ng/mL TGF-β1 for 72 h. The expression of *Oc* (A), *Osx* (B), and *Runx2* (C) mRNA was then measured. Figures shown represent at least three independent experiments. Values represent mean ± S.E. (*n* = 3). Bonferroni correction for multiple comparisons was applied. *, *p*<0.001; **, *p*<0.01; ***, *p*<0.05. (D) Cells were cultured for 2 weeks in OBM with or without TGF-β1. Mineralized matrix was visualized by Alizarin Red staining of cells.

Both *Oc and Osx* were expressed at significantly higher levels in CA-Akt cells than in Mock cells, but repeated TGF-β1 treatment suppressed this effect (Fig. 5A and B). *Runx2* expression in Mock cells was increased in the presence of TGF-β1, and expression of CA-Akt slightly increased *Runx2* mRNA levels (Fig. 5C). However, repeated TGF-β1 treatment abrogated this effect.

Mock cells cultured for 2 weeks in OBM showed slightly increased mineralization levels following a single treatment with 0.1 ng/mL TGF-β1; however, decreased mineralization was observed with repeated TGF-β1 treatment. For CA-Akt cells, significantly increased mineralization was observed under all medium conditions (Fig. 5D).

## Discussion

TGF-β1 exerts a range of effects on osteoblasts dependent on their maturity, culture conditions, and population density [33]. These different effects have been attributed to complex interactions between signaling cascades and their regulatory systems [24], [34]. The mechanisms underlying TGF-β1-induced osteoblast differentiation have been reported [35], [36]; however, the involvement and influence of other signaling pathways remain to be determined. We have previously reported that repeated TGF-β1 treatment could inhibit osteoblastic differentiation of HPDL cells because of decreased IGF-1 expression [30]. Consistently, TGF-β1 and IGF-1 are released from the bone tissue matrix and are responsible for critical events in bone regeneration. We have also shown that the IGF-1/PI3K pathway is indispensable for TGF-β1-induced osteogenesis [30]. In this current study, we aimed to elucidate the main pathway by which PI3K influences osteogenesis, and found that Akt activation could reverse the inhibitory effects of TGF-β1 on ALP expression in the murine osteoblast precursor cell MC3T3-E1.

MC3T3-E1 cells have been previously used to study the effects of TGF-β1 in osteoblast development [34], [37], and it has been established that TGF-β1 inhibits osteoblast differentiation of MC3T3-E1 cells. In this study, both the PI3K inhibitor LY294002 and repeated TGF-β1 treatment effectively inhibited both ALP activity and the mRNA expression of osteoblast markers in MC3T3-E1 cells.

Akt is a serine-threonine kinase and a major target molecule of PI3K [22], [23]. Therefore, we investigated whether constitutively activated Akt could reverse the inhibition of osteoblast differentiation induced by repeated TGF-β1 treatment. Although ALP activity was reduced in both Mock and DN-Akt cells following repeated treatment with TGF-β1, the ALP activity in CA-Akt cells was actually elevated. This indicates that TGF-β1 is able to enhance ALP activity in MC3T3-E1 cells, as long as Akt is activated. Exogenous IGF-1 administration can reverse the inhibition of osteoblast differentiation induced by repeated administration of TGF-β1 to MC3T3-E1 cells. Chen et al. identified that bone resorption supernatant, which reportedly includes IGF-1 and TGF-β1, could enhance Akt phosphorylation and promote the differentiation of MC3T3-E1 cells [38]. This is consistent with our findings that IGF-1 is required for TGF-β1-induced osteoblast differentiation. Inhibition of Akt phosphorylation by use of a DN-Akt could not reverse the suppression induced by TGF-β1 even with IGF-1 treatment. These results indicate that TGF-β1 regulates osteoblast differentiation via IGF-1/PI3K/Akt. In the present study, it is demonstrated that TGF-β1 could decrease the expression of *Alp* and *Oc* in DN-Akt cells. This finding may indicate that other signaling pathways such as Smad and MAPK are likely to be involved in this process.

*Oc* mRNA levels are a marker of terminal osteoblast differentiation [39], and were decreased depending on the number of TGF-β1 administrations in both Mock and CA-Akt cells. Mock cells also underwent decreased mineralization with repeated administration of TGF-β1. Similarly, repeated TGF-β1 treatments inhibited mineralization of CA-Akt cells, although basal mineralization was increased in these cells. Collectively, these results indicate that TGF-β1 enhances osteoblast differentiation via activation of Akt in the early phase of differentiation rather than in the late phase. Compared with Mock cells, the degree of mineralization was significantly increased by CA-Akt in all medium conditions. In CA-Akt cells, TGF-β1 promoted osteoblast differentiation by increasing ALP expression. Akt is thought to be essential for mineralization based on the evidence that Akt1/Akt2 knockout mice exhibit delayed bone ossification [40], and that Akt1 KO mice showed reduced bone size and delayed formation of secondary ossification centers [41]. In this study, cells expressing CA-Akt had elevated *Oc* levels in all medium conditions when compared with Mock cells. This suggests that phosphorylation of Akt is essential for osteoblast differentiation. However, interestingly, expression of the late phase osteoblast markers (*Osx*, *Oc*) was decreased as the number of TGF-β1 treatments increased. This indicates that Akt activation alone is not sufficient to reverse the inhibitory effect of TGF-β1 on the late phase of osteoblast differentiation.

Some studies have reported that crosstalk between TGF-β1 and other signaling pathways exists during osteoblast differentiation [24], [34]. A relationship between Akt and TGF-β1 has also been demonstrated in previous studies [6], [42], while PDGF positively modulates TGF-β-induced osteogenic differentiation of human mesenchymal stem cells through synergistic crosstalk between MEK and PI3K/Akt signaling [43]. Furthermore, Remy et al. have shown that Akt inhibits the transcriptional activity of Smad3 by direct binding in cancer cells [44]. In the present study, western blot analysis showed that CA-Akt enhanced the phosphorylation of Erk1/2 and Smad3 (Fig. S1). Our results indicate that CA-Akt did not interfere with either the canonical or non-canonical pathway of TGF-β1, but rather influenced TGF-β1 signaling and enhanced TGF-β1-induced osteogenesis.

In conclusion, our results reveal that activation of Akt positively regulates the early phase of TGF-β1-induced osteoblast differentiation as evidenced by increased ALP expression following TGF-β1 treatment. Although activation of Akt enhanced the late phase of osteoblast differentiation with increased *Oc* expression and mineralization, repeated TGF-β1 treatments decreased *Oc* expression even in the presence of CA-Akt expression. Thus, PI3K/Akt signaling differentially regulates TGF-β1 signaling at different stages of osteoblast differentiation. Further studies are needed to clarify the mechanisms responsible for this.

TGF-β1 plays a major role in inflammatory conditions [5], and high TGF-β1 production is considered to be a protective factor for periodontitis [45]. Although a pivotal role in the bone-remodeling process has been assigned to TGF-β1 [7], excessive TGF-β1 production can also inhibit osteoblast differentiation. We have now shown that PI3K/Akt signaling plays an important role in reversing the inhibition of osteoblastic differentiation by TGF-β1. Our results indicate that Akt is the molecular switch for TGF-β1-induced osteoblastic differentiation of MC3T3-E1 cells. The activation of Akt could therefore be a useful treatment approach for bone regeneration in inflammatory disease.

## Supporting Information

Figure S1 **Akt activation enhances phosphorylation of Erk1/2 and Smad3.** MC3T3-E1 cells were infected with CA-Akt vector or Mock vector, and then cells were treated with or without repeated administration of 0.1 ng/mL TGF-β1 for 72 h. Protein was extracted and analyzed by western blot. Antibodies used were anti-Erk1/2, anti-phosphorylated Erk1/2, anti-Smad3, anti-phosphorylated Smad3, and β-Actin (all 1∶1000, except anti-Erk1/2 1∶2000; all from Cell Signaling Technology Inc.).
(TIF)
