# Supplementary figures and images for: Akt Activation is Required for TGF-β1-Induced Osteoblast Differentiation of MC3T3-E1 Pre-Osteoblasts
Source: PLoS One. 2014 Dec 3;9(12):e112566. doi: 10.1371/journal.pone.0112566 (PMC4254279; doi:10.1371/journal.pone.0112566)

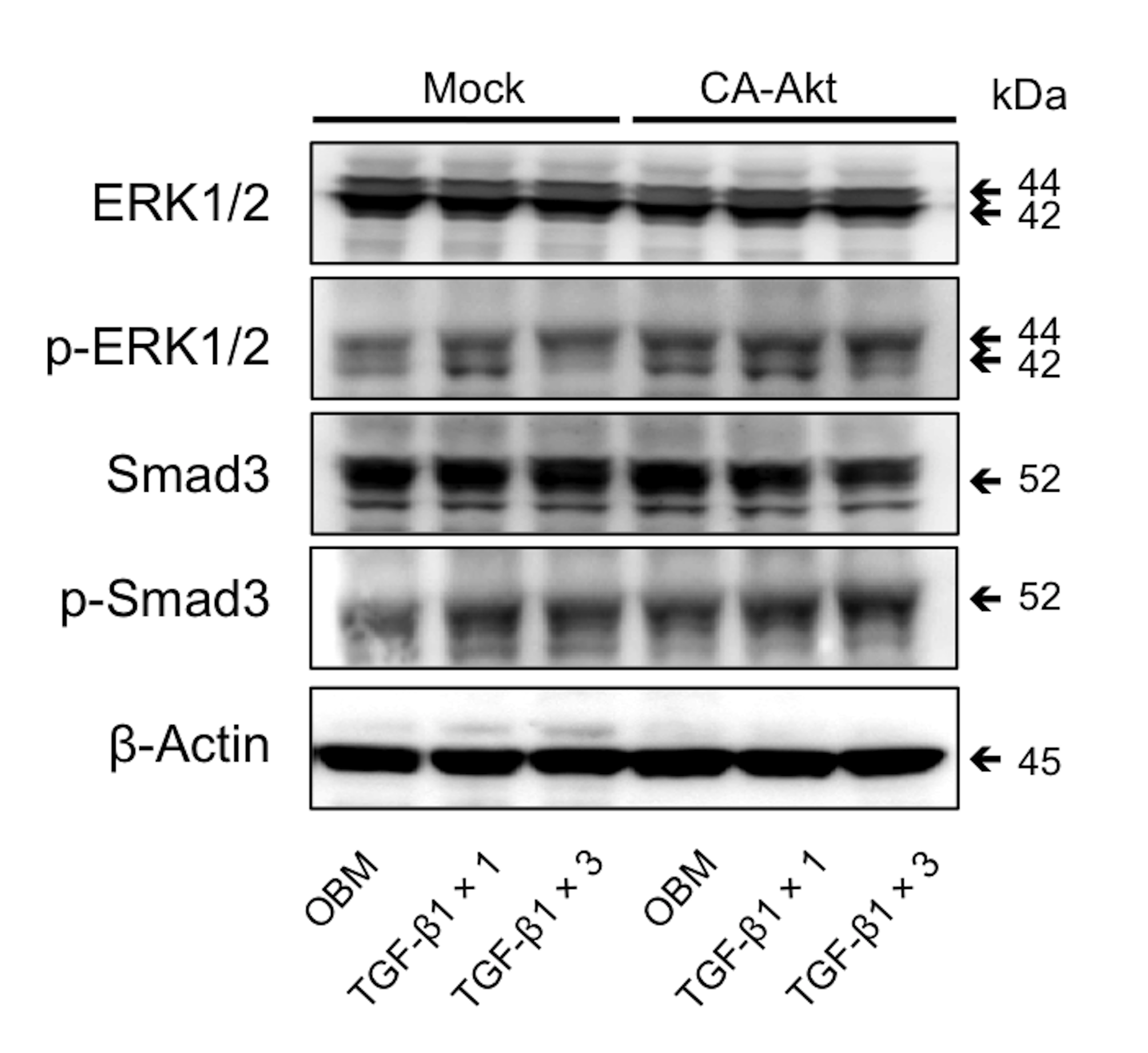

Supplement: Figure S1 — Akt activation enhances phosphorylation of Erk1/2 and Smad3. MC3T3-E1 cells were infected with CA-Akt vector or Mock vector, and then cells were treated with or without repeated administration of 0.1 ng/mL TGF-β1 for 72 h. Protein was extracted and analyzed by western blot. Antibodies used were anti-Erk1/2, anti-phosphorylated Erk1/2, anti-Smad3, anti-phosphorylated Smad3, and β-Actin (all 1∶1000, except anti-Erk1/2 1∶2000; all from Cell Signaling Technology Inc.). (TIF) [file pone.0112566.s001.tif]
